# Evaluation of the effect of cell freshness on pyrogen detection using a serum-free monocyte-activation test

**DOI:** 10.1371/journal.pone.0316203

**Published:** 2024-12-30

**Authors:** Katsuhiko Hayashi, Mizuki Sano, Toshie Kanayasu-Toyoda, Yuji Morita, Teruhide Yamaguchi, Kenji Ohya, Yutaka Kikuchi, Ken-ichi Izutsu, Yukiko Hara-Kudo

**Affiliations:** 1 Division of Microbiology, National Institute of Health Sciences, Kawasaki, Kanagawa, Japan; 2 Department of Infection Control Science, Meiji Pharmaceutical University, Kiyose, Tokyo, Japan; 3 Department of Pharmaceutical and Medical Business Sciences, Nihon Pharmaceutical University, Kitaadachi-gun, Saitama, Japan; 4 Institute of Advanced Medical and Engineering Technology for Aging, Kanazawa Institute of Technology. Nonoichi, Ishikawa, Japan; 5 Department of Nutrition, Faculty of Healthcare Sciences, Chiba Prefectural University of Health Sciences, Chiba, Japan; 6 Department of Pharmaceutical Sciences, School of Pharmacy International University of Health and Welfare, Ohtawara, Tochigi, Japan; 7 Division of Animal Life Science, Institute of Agriculture, Tokyo University of Agriculture and Technology, Fuchu, Tokyo, Japan; Universidad Nacional de la Plata, ARGENTINA

## Abstract

Pyrogens cause shock symptoms when released into the bloodstream. They are classified into two main categories: endotoxins (lipopolysaccharides [LPS]) and non-endotoxin pyrogens. The monocyte activation test (MAT) is an *in vitro* assay to detect pyrogens in human monocytes. Cells were incubated in the culture medium, and the cellular response, specifically the production of the inflammatory cytokine interleukin-6 in the culture supernatant, was analyzed using enzyme-linked immunosorbent assay (ELISA). Technical improvements, such as cell acquisition and culture media selection, will be beneficial for the popularization of MAT. The cell freshness was strictly controlled to achieve high MAT sensitivity. However, it is necessary to investigate the usability of older and stored blood samples in the MAT. This study evaluated the effect of cell freshness on MAT using peripheral blood mononuclear cells (PBMCs) isolated from 2- and 5-d-old donated whole blood samples. To mitigate the influence of serum in the culture medium, a serum-free MAT was developed using the LPS-binding protein (LBP) as an enhancer for LPS detection. PBMCs were incubated with a two-fold dilution series of LPS at 0.001–4.096 endotoxin units/mL (EU/mL). Interleukin-6 levels in the culture supernatant were quantified by ELISA in the presence and absence of LBP. In the presence of LBP, the limit of detection (LOD) for LPS was 0.001–0.008 EU/mL. However, in the absence of LBP, the LOD was 0.512 EU/mL. Peripheral PBMCs were 38.6 times more sensitive in the presence of LBP than in its absence. When utilizing the developed serum-free MAT with LBP, 5-d-old PBMCs showed LODs of 0.016–0.064 EU/mL, indicating a 3.1-fold increase in sensitivity compared with 5- to 2-d-old PBMCs. These results suggest that the sensitivity of PBMCs decreased gradually rather than sharply. The study concluded that 2-d-old PBMCs were sufficiently fresh and could be used as serum-free MAT.

## Introduction

Pyrogens, which can elicit shock symptoms when released into the bloodstream, are classified into two main categories: endotoxins (lipopolysaccharides [LPS]) and non-endotoxin pyrogens (NEPs) [1]. LPS, a component of the outer membrane of gram-negative bacteria, is the most significant pyrogen [2]. The latter group includes a variety of substances, such as bacterial flagellin [3], cell wall components of gram-positive bacteria [4], viral RNA [5], and bacterial DNA [6]. Pyrogens are traditionally detected using the bacterial endotoxin test (BET) and rabbit pyrogen test (RPT) to prevent the distribution of pyrogen-contaminated pharmaceutical injections [7, 8]. The BET assay specifically detects LPS, whereas the RPT assay detects both LPS and NEPs. Despite its usefulness, RPT sometimes produces false negatives [9], raising ethical concerns because of the use of animals [10]. As an alternative to the RPT, a monocyte-activation test (MAT), which uses human blood or cells, has been developed [1, 10, 11].

According to the MAT procedures described in Chapter 2.6.30 of the European Pharmacopoeia (Ph. Eur.) [12], human blood, peripheral blood mononuclear cells (PBMCs), and monocytic cell lines at a final concentration of 0.1–1.0 × 10^6^ cells/mL were cultured in media containing either the donor’s own plasma, human AB serum or foetal bovine serum. During culture, monocytic cells recognize LPS via toll-like receptor 4 (TLR4) with the assistance of LPS-binding protein (LBP) in the serum or plasma [13, 14]. NEPs have also been identified in other TLRs. For example, cell wall components of gram-positive bacteria, including heat-killed *Staphylococcus aureus* (HKSA), bind to TLR2 complexes [4, 15]. After culturing, the released inflammatory cytokines, such as interleukin-6 (IL-6), were measured using an enzyme-linked immunosorbent assay (ELISA).

Chapter 2.6.30, Ph. Eur. [12] recommends that MAT cells should be used for no more than 4 h after collection; however, such fresh cells are unavailable in some countries, including Japan. The Japanese Red Cross Society (JRCS) provides donated whole blood products unsuitable for therapeutic use (out-of-specification, such as insufficient volume) at least 2-d-old. Additionally, Ph. Eur. recommended that culture media be supplemented with serum, which may contain interfering factors such as LPS and other pyrogens. To mitigate the influence of the serum, a trial on serum-free MAT was recently reported [16]. Technical improvements in cell acquisition and culture media selection are beneficial for the broader adoption of MAT.

To achieve good MAT sensitivity, cell freshness was strictly controlled in a verification study by the Interagency Coordinating Committee on the Validation of Alternative Methods (ICCVAM) [17]. Three MAT tests [18–20] using freshly drawn blood and freshly prepared PBMCs detected low levels of LPS (0.042–0.082 endotoxin units/mL [EU/mL]) in the culture medium. In addition, MAT tests [21, 22] using human blood and PBMCs cryopreserved immediately after donation detected 0.002–0.042 EU/mL of LPS in the culture medium. However, detailed investigations on the usability of older blood samples stored under refrigeration for MAT are lacking. An improved understanding of the effect of cell freshness on the MAT can aid in designing more efficient testing procedures.

This study aimed to determine the effects of cell freshness on MAT. To mitigate the effects of serum, a serum-free MAT was developed using LBP as an enhancer for LPS detection. HKSA and LPS were used to evaluate the serum-free MAT. In the serum-free MAT, PBMCs isolated from 2- and 5-d-old donated whole blood were cultured in a serum-free medium. After culturing, the amount of IL-6 in the culture supernatant was measured using ELISA, and the limit of detection (LOD) was determined. The LOD and reactivity to LPS were analyzed by comparing the results of 2- and 5-d-old PBMCs to clarify their effects on freshness.

## Materials and methods

### Ethics statement

The study design and protocol for the use of donated blood were revised and approved by the Institutional Review Boards of the National Institute of Health Sciences, Japan (approval no. 341, from August 3, 2020, to March 31, 2025) and the JRCS (approval no. R030028, from March 26, 2021, to March 31, 2024). All participants (adults) provided written informed consent before blood donation, and whole blood was purchased from the JRCS according to the *Guidelines on the Use of Donated Blood in Research and Development*, *etc*. Donated blood was only provided when insufficient amounts of blood-derived products (out-of-specification) were found. The purchase dates of donated blood for PBMCs lots were as follows: #D2.1 on November 10, 2021; #D2.2 on December 14, 2021; #D2.3 on December 14, 2021; #D2.4 on December 1, 2021; #D2.5, #D2.6, and #D2.7 on December 19, 2023; #D2.8 on September 8, 2022; #D5.01–04 on October 22, 2021; #D5.05–08 on October 22, 2021; #D5.09–12 on November 18, 2021; and #D5.13–16 on November 18, 2021.

### Preparation of LPS

The Japanese Pharmacopoeia reference standard endotoxin (Pharmaceutical and Medical Device Regulatory Science Society of Japan, Osaka, Japan) was used to prepare LPS, which was dissolved in sterile distilled water (Otsuka Pharmaceutical Factory, Tokushima, Japan) and vortexed for 5 min to prepare a 10, 000 EU/mL stock solution.

### Preparation of HKSA

PANSORBIN cells (Merck KGaA, Darmstadt, Germany) [22–24], which were heat-killed, formalin-fixed *S*. *aureus*, were used. Additionally, a homemade HKSA was used. For the homemade HKSA preparation, *S*. *aureus* strain NBRC 13276 (National Institute of Technology and Evaluation Biological Resource Center, Chiba, Japan) was cultured at 30–35°C in Dulbecco’s Modified Eagle Medium (MEM)/Nutrient Mixture F-12 with 4-(2-hydroxyethyl)-1-piperazineethanesulfonic acid and no phenol red (Nacalai Tesque, Kyoto, Japan) containing 2× Basal Medium Eagle vitamins (Merck KGaA, Darmstadt, Germany), 2× MEM Essential Amino Acid Solution (Fujifilm Wako Pure Chemical Corporation, Osaka, Japan), and 10× Non-essential Amino Acid Solution (Fujifilm Wako Pure Chemical Corporation). The culture was passed through a glass filter (GF/C; Cytiva, Marlborough, MA, USA). The bacterial body was washed twice with sterile normal saline (Otsuka Pharmaceutical Factory, Tokushima, Japan) and heated at 100°C for 10 min to prepare the HKSA stock solution. The HKSA cells were counted using a bacterial counter (Sunlead Glass, Saitama, Japan).

### Preparation of pyrogen panel plates

The LPS stock solution was diluted from 0.02 to 327.68 EU/mL in a two-fold series, resulting in a 20-fold incubation concentration series (0.001–16.384 EU/mL). Similarly, the HKSA stock solutions were diluted in a two- or 0.5-log_10_-fold series. Ten microliters of each dilution were distributed into each well in quadruplicate on a culture-treated 96-well plate (AGC Techno Glass, Shizuoka, Japan).

### Preparation of the PBMCs from donated blood

Samples of 2-d-old whole blood (out-of-specification products because of insufficient amounts; sample volume: 240–400 mL) and 5-d-old residual whole blood (test specimens; sample volume: approximately 4 mL) under refrigeration were purchased from the JRCS. To isolate PBMCs, whole blood samples were centrifuged at 1,750 × *g* for 10 min at 18°C to remove blood clots, and the PBMC-rich fraction was collected. This fraction was diluted to twice the volume with sterile phosphate-buffered saline (PBS (−), Fujifilm Wako Pure Chemical Corporation, Osaka, Japan), containing 2 mmol/L ethylenediaminetetraacetic acid (sPBS-EDTA) pH 8.0 (Nippon Gene, Tokyo, Japan) to avoid the aggregation of PBMCs. The mixture was layered onto a Lymphoprep solution (Serumwerk, Bernburg, Germany) and centrifuged at 1,000 × *g* for 20 min at 18°C to separate the PBMCs. The layer was diluted to three times the volume with sPBS-EDTA and centrifuged again at 1,000 × *g* for 10 min at 4°C. The collected PBMCs were washed twice with 10 mL of sPBS-EDTA and suspended in X-VIVO15 serum-free medium (Lonza, Basel, Switzerland). Cells were stained with 0.2% trypan blue, and the number of live cells was counted using a TC10 automated cell counter (Bio-Rad, Hercules, CA, USA). The live cell number was adjusted to 1.6×10^6^ cells/mL using X-VIVO15 medium. For 5-d-old PBMCs, equal volumes from the four lots were mixed.

### Monocyte-activation test

To each well of the pyrogen panel plates, containing 10 μL of pyrogen per well, 190 μL of PBMCs were added at a final concentration of 8×10^5^ cells/mL in X-VIVO15 serum-free medium supplemented with a final concentration of 100 ng/mL of LBP (recombinant human LBP without bovine serum albumin; R&D Systems, Minneapolis, MN, USA). The mixture was cultured in a humidified atmosphere containing 5% CO_2_ at 36–38°C for 22–26 h. Following incubation, the culture supernatant was collected and used for ELISA.

### ELISA

The Human IL-6 ELISA Set (Diaclone, Besançon, France) was used according to the manufacturer’s instructions with minor modifications. The standards of IL-6 were prepared in duplicate with 100 μL of 1% bovine serum albumin in phosphate-buffered saline (0 pg/mL) and IL-6 solution (3.13, 6.25, 12.5, 25, 50, 100, and 200 pg/mL). For sample preparation, 25 μL of each supernatant was mixed with 75 μL of PBS(−) to prepare 4-fold diluted samples. For the detection, a 3,3’,5,5’-tetramethylbenzidine solution was used. After acidification with 1 mol/L sulfuric acid, the resultant yellow color was measured as the absorbance at 450 nm with a reference at 630 nm using an ELx808IU plate reader (Agilent Technologies, Santa Clara, CA, USA). The difference in absorbance between 450 and 630 nm was used for the statistical analysis.

### Statistical analysis

All statistical analyses were performed using Excel (Microsoft, Redmond, WA, USA) and R software version 4.3.0 (R Core Team 2023) [25].

To detect outliers, the quadruplicated results within a plate were evaluated using the Smirnov–Grubbs test with a significance level of *α* = 0.05. Only one outlier was detected and removed from the quadruplicate results; thus three or four values were analyzed.

The IL-6 concentration was determined using a standard curve, which was calculated using the ELISA results of the standard solutions using 4-parameter logistic regression. The mean concentration and standard deviation (SD) of IL-6 levels were also calculated.

To determine the LOD, the threshold was calculated, as described in Chapter 2.6.30. of the Ph. Eur [12]. Briefly, using the results without pyrogen (blank), a threshold was determined by adding the mean of the ELISA absorbance values, which reflected IL-6 production, to their SD multiplied by three (threshold = mean value [blank] + 3× SD [blank]). The threshold was converted to IL-6 levels using a standard curve of ELISA. The LOD was defined as the lowest concentration of LPS and HKSA, which cause a monotonically increased mean IL-6 concentration above this threshold. The statistical significance of the IL-6 concentration was evaluated by Welch’s *t*-test with α value of 0.05. To evaluate the accuracy of the assays, the coefficient of variation (CV) was calculated using the IL-6 values above LOD, by dividing the SD of IL-6 by the mean value of IL-6.

To compare the reactivity of the cells, linear regression and parallel-line analyses were performed using the logarithmic IL-6 concentrations and logarithmic LPS or HKSA concentrations above the LOD. Linear regression was performed to calculate the slope, intercept, and coefficient of determination (*R*^2^) values using the least-squares method. The linear region of the data was determined to have a good correlation when the *R*^2^ value was 0.5 or higher (*R*^2^ > 0.5). Using the linear region of the data, a parallel-line analysis was performed by analysis of variance using an *F*-test at a significance level of *p* < 0.05. If parallelism was rejected, the differences in reactivity were calculated using the mean LOD and middle of the exponential phase of the data. If parallelism was not rejected, differences were calculated using a common slope.

To evaluate the assay reproducibility, the equivalent LPS amount (equivalent endotoxin unit [EE]) of HKSA was calculated using the relationship between IL-6 and LPS concentrations. The IL-6 concentrations in the blanks and samples above LOD from MAT for LPS were analyzed using 4-parameter logistic regression. This regression was used as a calibration curve to convert from IL-6 concentrations to EE values. IL-6 concentrations above the LOD from the MAT for HKSA were converted to EE values (EE/mL). The EE values from the MAT assay were divided by the corresponding HKSA concentrations, and the divided values were averaged to calculate the EE value of HKSA (EE/cells). The mean ± SD of EE values of HKSA from MAT assays was also calculated.

## Results

### LPS detection by serum-free MAT using 2-d-old PBMCs in the presence and absence of LBP

**Fig 1:**
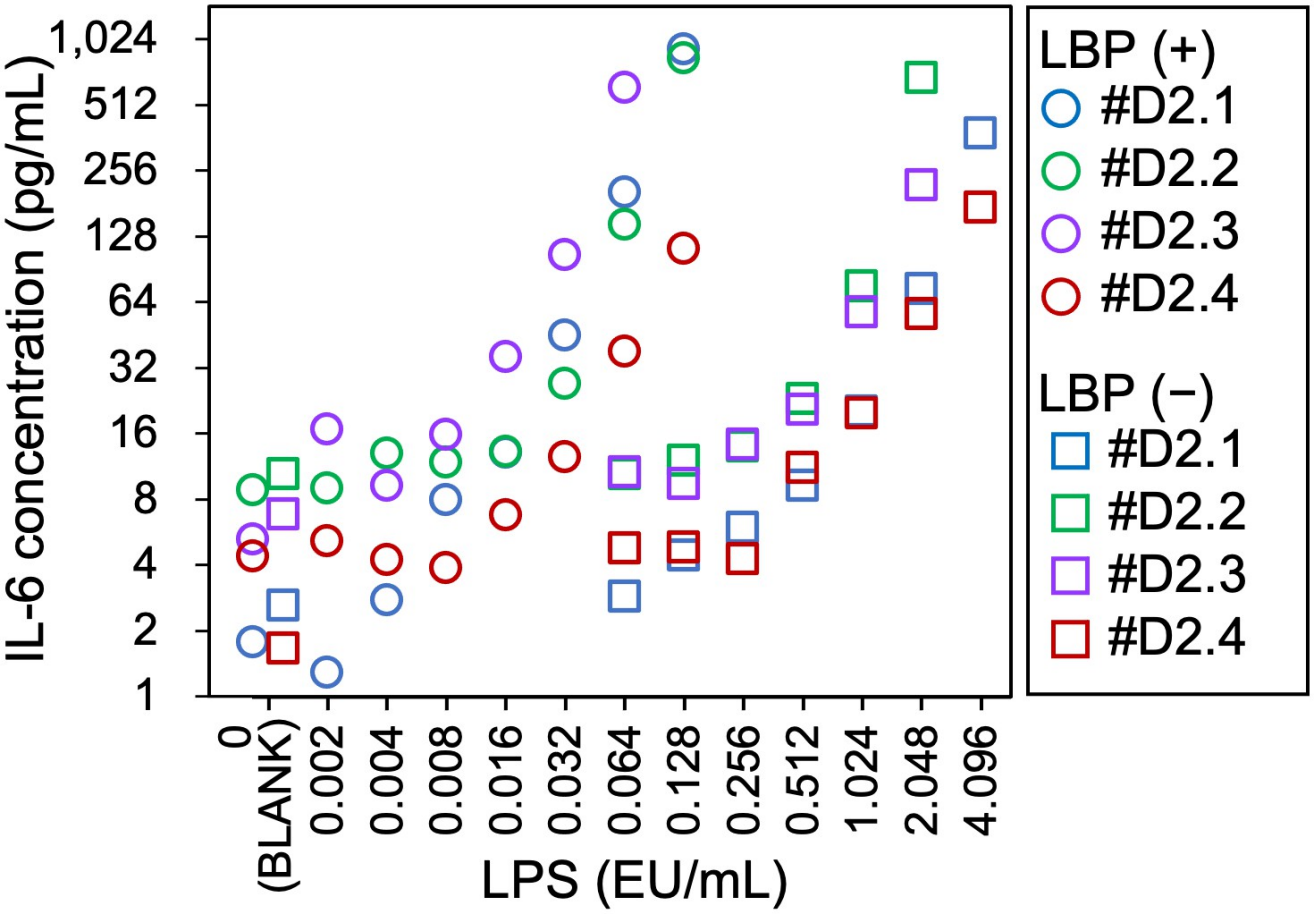
Detection of lipopolysaccharide (LPS) by serum-free monocyte-activation tests using 2-d-old peripheral blood mononuclear cells (PBMCs) in the presence of LPS-binding protein (LBP). The mean interleukin-6 (IL-6) levels in the culture supernatant of the four PBMC lots (#D2.1–D2.4) were plotted. LBP (+), in the presence of 100 ng/mL of LBP; LBP (−), in the absence of LBP; EU, endotoxin unit.

The mean IL-6 levels of four lots of 2-d-old PBMCs (#D2.1, #D2.2, #D2.3, and #D2.4) in the presence or absence of LBP were plotted in Fig 1 (all statistical details were in S1 File). Increases in IL-6 levels depended on LPS concentration, regardless of the presence or absence of LBP; however, the range of LPS concentrations causing the increase in the presence of LBP was lower than that in the absence of LBP. In the presence of LBP, the four PBMC lots #D2.1, #D2.2, #D2.3, and #D2.4 showed that their LODs were 0.008, 0.032, 0.008, and 0.008 EU/mL, respectively (S1 Fig and S1 File). IL-6 concentrations at these LODs were significantly higher than the IL-6 concentration in the blank (0 EU/mL) tests, with *p*-values of 0.019, 0.017, <0.001, and <0.001 for PBMC lots #D2.1, #D2.2, #D2.3, and #D2.4, respectively. In the absence of LBP, all four lots exhibited an LOD of 0.512 EU/mL (S2 Fig and S1 File). IL-6 concentrations at these LODs were also significantly higher than those in the blank tests, with *p*-values of 0.002, <0.001, 0.014, and 0.004 for PBMC lots #D2.1, #D2.2, #D2.3, and #D2.4, respectively.

**Fig 2:**
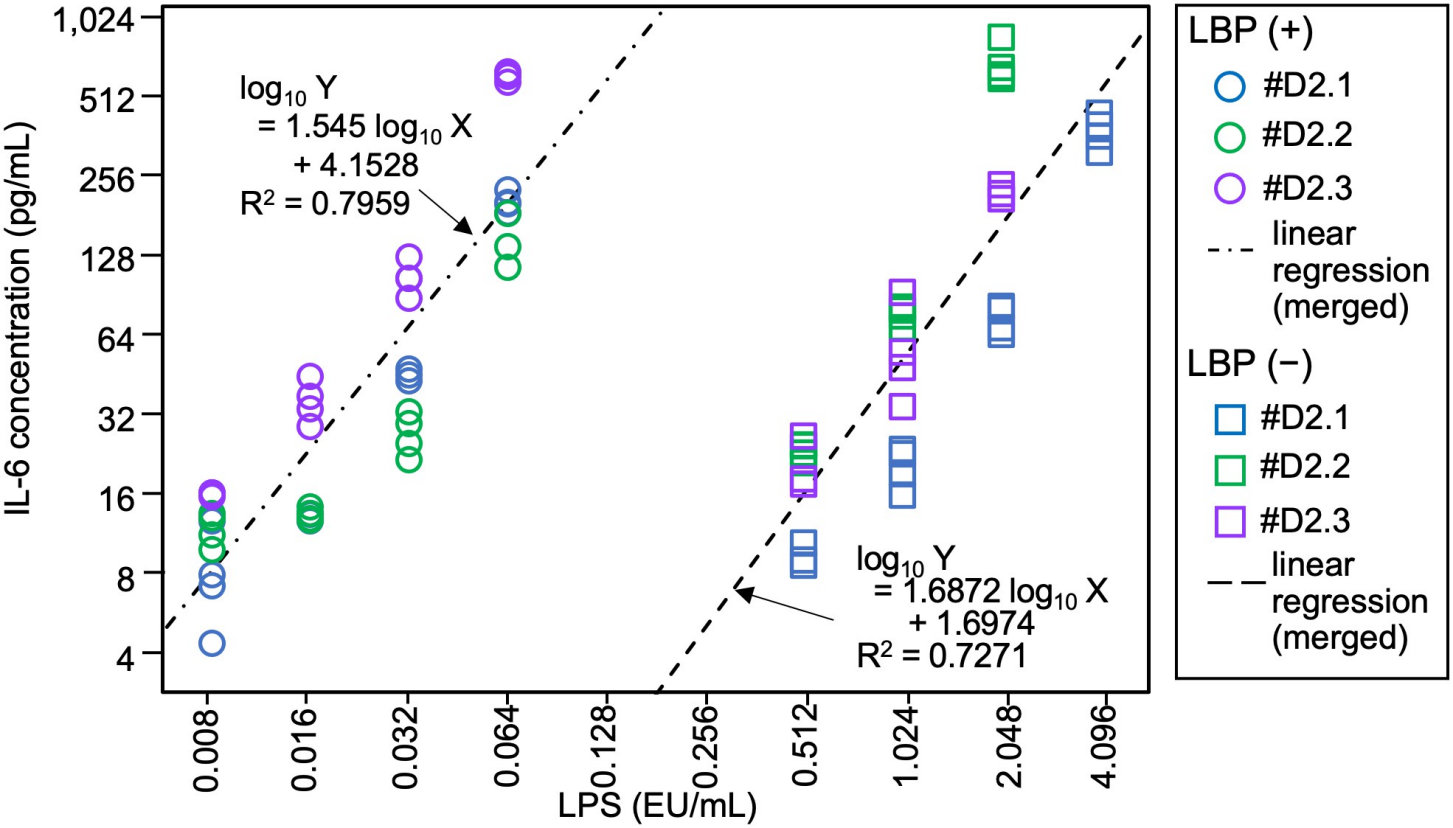
Comparison of the reactivities to lipopolysaccharide (LPS) on the serum-free monocyte-activation tests in the presence and absence of LPS-binding protein (LBP). Using the data from the three PBMC lots (#D2.1–3), the interleukin-6 (IL-6) production in culture supernatant above the limit of detection was plotted. Dash-dot and short-dashed lines represent linear regressions using the merged data in the presence and absence of LBP, respectively. Next to those regression lines, the regression equations are presented. LBP (+), in the presence of 100 ng/mL of LBP; LBP (−), in the absence of LBP; EU, endotoxin unit.

To compare the reactivities of serum-free MAT in the presence and absence of LBP, parallel-line analysis was performed using the IL-6 levels from three of four lots (#D2.1, #D2.2, and #D2.3) in the presence or absence of LBP (Fig 2 and S1 File) because the data from lot #D2.4 showed a relatively higher LOD (S1 Fig). Linear regressions for the merged data of three PMBC lots in the presence and absence of LBP yielded two lines with *R*^2^ values of 0.7959 and 0.7271, respectively. An *F*-test indicated that these lines are parallel, with a *p*-value of 0.988. A 38.6-fold increase in reactivity was observed in the presence and absence of LBP. Parallel-line analysis of each lot (#D2.1, #D2.2, #D2.3, and #D2.4), comparing the absence to the presence of LBP, showed a 50.9-fold, 11.9–34.3-fold, 51.2-fold, and 17.7–24.9-fold increase, respectively (S3 Fig and S1 File).

### HKSA detection by serum-free MAT

The S4 Fig illustrates the IL-6 levels in three lots of 2-d-old PBMCs (#D2.5, #D2.6, and #D2.7) utilizing HKSA samples. Both the HKSA-PANSORBIN cells and homemade HKSA cells exhibited comparable trends, demonstrating a dependency on HSKA concentration, which increased IL-6 levels. The LODs of HKSA-PANSORBIN cells using three lots of 2-d-old PBMCs (#D2.5, #D2.6, and #D2.7) were 1.0×10^4^, 3.2×10^4^, and 3.2×10^4^ cells/mL, with *p*-values of 0.003, 0.009, and <0.001, respectively (S4A–S4C Fig and S1 File), corresponding to 0.0000041–0.000013% of the original PANSORBIN cell solution. The LODs of the homemade HKSA using PBMC lots #D2.5, #D2.6, and #D2.7 were 1.2×10^4^, 3.8×10^4^, and 1.2×10^5^ cells/mL, with *p*-values of 0.031, 0.007, and 0.011, respectively (S4D–S4F Fig and S1 File).

In the presence or absence of LBP, a parallel analysis was performed on IL-6 levels in 2-d-old PBMCs (lot #D2.8) using homemade HKSA (S5 Fig and S1 File). The difference in reactivity showed a 1.3-fold increase in the presence of LBP with almost no increase in reactivity. It should be noted that in the presence of LBP, LODs of LPS using 2-d-old PBMC lots #D2.5, #D2.6, #D2.7, and #D2.8 were 0.001, 0.008, 0.002, and 0.008 EU/mL, with *p*-values of 0.007, 0.009, <0.001, and <0.001, respectively (S6 Fig and S1 File).

### LPS detection by serum-free MAT using 5-d-old PBMCs

**Fig 3:**
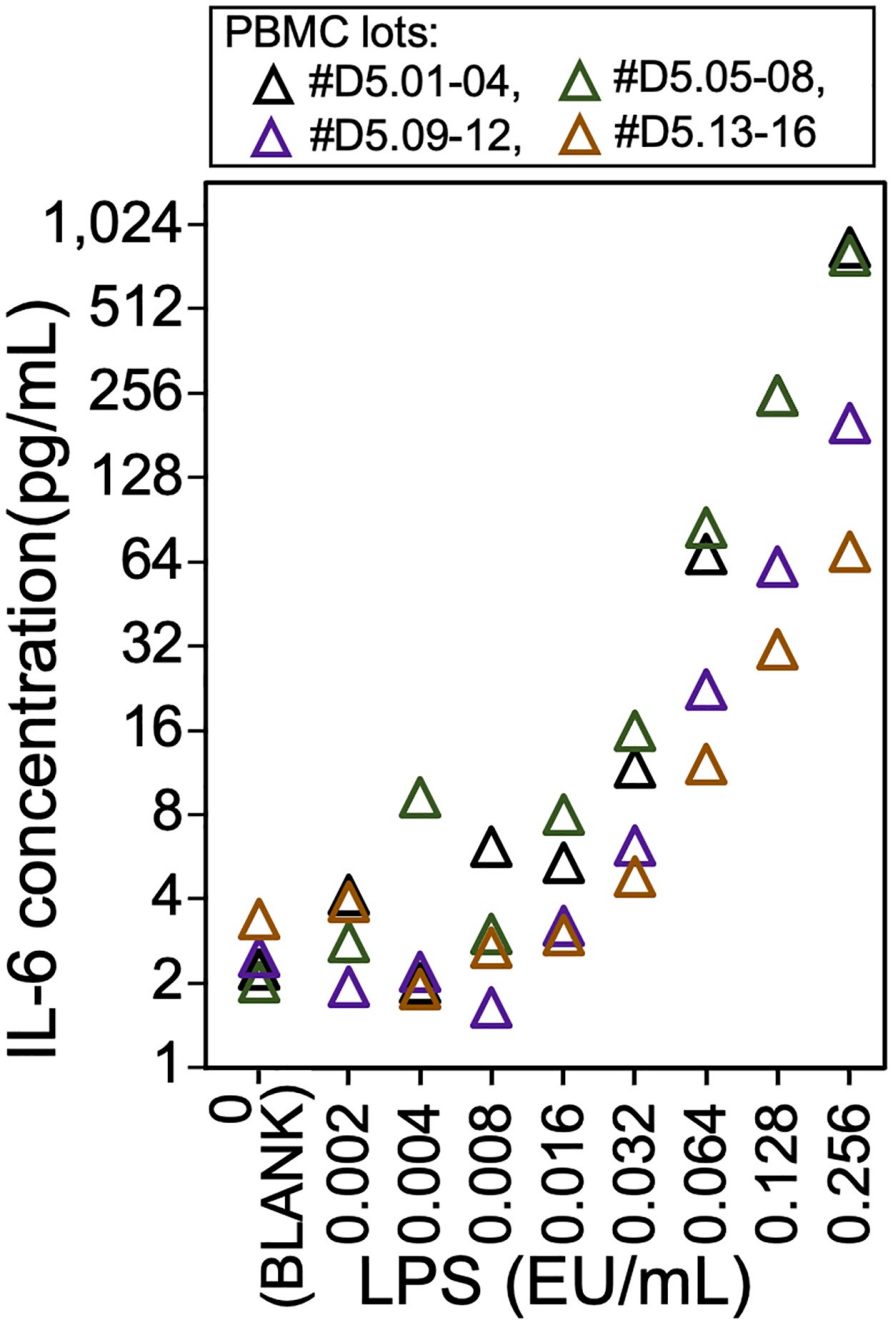
Detection of lipopolysaccharide (LPS) by serum-free monocyte-activation test using 5-d-old peripheral blood mononuclear cells (PBMCs) in the presence of LPS-binding protein (LBP). The mean interleukin-6 (IL-6) levels in the culture supernatant of four experiments using 16 lots of PBMCs (#D5.01–04, #D5.05–08, #D5.09–12, and #D5.13–16) were plotted. EU, endotoxin unit.

MAT in the presence of LBP was performed in four independent experiments using 16 lots of 5-d-old PBMCs (#D5.01–04, #D5.05–08, #D5.09–12, and #D5.13–16). IL-6 levels are plotted in Fig 3 (all statistical details were in S1 File). All experiments showed consistent dependence with an increase in IL-6 levels with increasing LPS concentrations. The LODs of the four experiments were 0.016, 0.016, 0.032, and 0.064 EU/mL, with *p*-values of 0.007, 0.003, 0.001, and 0.002, respectively (S7 Fig and S1 File).

**Fig 4:**
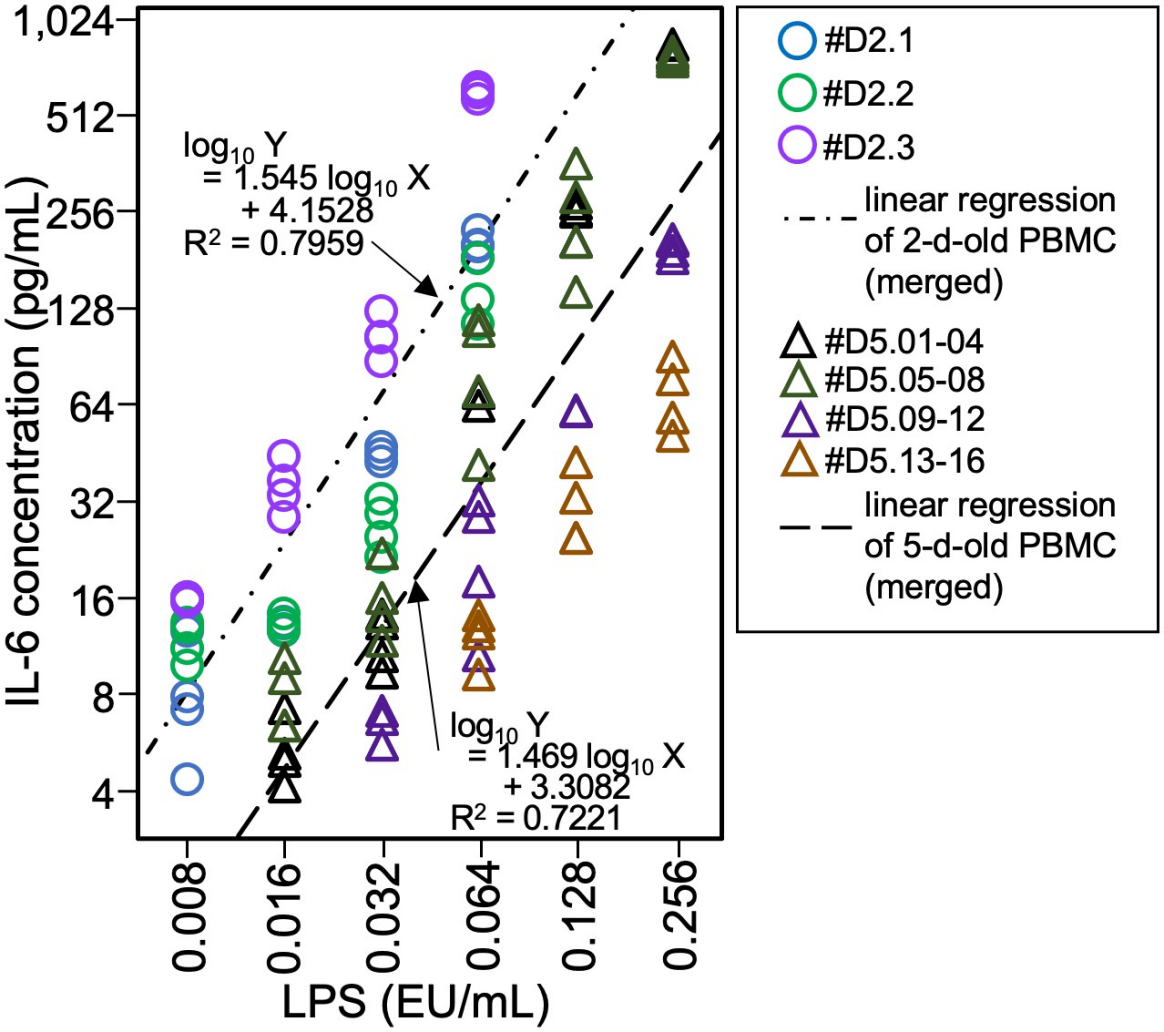
Comparison of the reactivities to lipopolysaccharide (LPS) on serum-free monocyte-activation tests using 2- and 5-d-old peripheral blood mononuclear cells (PBMCs). Using the data from three lots of 2-d-old PBMCs (#D2.1–3) and four experiments of 5-d-old using 16 lots of PBMCs (#D5.01–04, #D5.05–08, #D5.09–12, and #D5.13–16), the interleukin-6 (IL-6) production in culture supernatant above the limit of detection was plotted. Dash-dot and long-break lines represent the linear regressions using the merged data of 2- and 5-d-old PBMCs, respectively. Next to those regression lines, regression equations are provided. EU, endotoxin unit.

To compare the reactivities of serum-free MAT using 2- and 5-d-old PBMCs, parallel-line analysis was performed using the IL-6 levels of 2-d-old PBMCs lots #D2.1–3 and four independent experiments of 5-d-old PBMCs lots #D5.01–04, #D5.05–08, #D5.09–12, and #D5.13–16 (Fig 4 and S1 File). Linear regressions for the merged data of 5-d-old PBMCs yielded a line with an *R*^2^ value of 0.7221. When comparing 5- and 2-d-old PBMCs in the presence of LBP, an *F*-test indicated that the two lines are parallel, with a *p*-value of 1.000. There was a 3.1-fold increase, and the range of LPS concentrations for the increase in IL-6 levels was similar.

### Accuracy and reproducibility of the serum-free MAT with LBP

To evaluate the accuracy of the serum-free MAT with LBP, the CV was calculated at each concentration of LPS and HKSA above the LOD (Table 1 and S1 File). Of the 59 data points for 2-d-old PBMCs, 51 (86%) had a CV of < 20% (51/59). Regarding CVs below 30%, 57 of the 59 data points (97%) met the criterion. Of the 17 data points for 5-d-old PBMCs, nine (53%) had a CV of < 20% (9/17). The CV values tended to increase near LOD.

**Table 1:** Coefficients of variation for monocyte-activation test assays.

LPS (EU/mL)	PBMC Lot	#D2.1	#D2.2	#D2.3	#D2.4	#D2.5	#D2.6	#D2.7	#D2.8	#D5. 01–04	#D5. 05–08	#D5. 09–12	#D5. 13–16
0.002		-	-	-	-	24%	-	5.3%	-	-	-	-	-
0.004		-	-	-	-	16%	-	13%	-	-	-	-	-
0.008		43%	14%	1.8%	-	17%	20%	17%	13%	-	-	-	-
0.016		3.5%	5.6%	18%	-	13%	15%	19%	18%	25%	25%	-	-
0.032		5.2%	18%	15%	4.7%	4.7%	10%	20%	11%	19%	28%	13%	-
0.064		8.1%	24%	3.9%	12%	-	-	4.2%	8.2%	5.1%	42%	45%	17%
0.128		5.4%	6.8%	-	7.8%	-	-	-	-	12%	38%	0.3%	27%
0.256		-	-	-	-	-	-	-	-	3.2%	2.9%	6.1%	26%
Homemade HKSA (cells/mL)	PBMC Lot	#D2.1	#D2.2	#D2.3	#D2.4	#D2.5	#D2.6	#D2.7	#D2.8	#D5. 01–04	#D5. 05–08	#D5. 09–12	#D5. 13–16
1.2×10^4^		-	-	-	-	30%	-	-	-	-	-	-	-
3.8×10^4^		-	-	-	-	17%	20%	-	-	-	-	-	-
4.5×10^4^		-	-	-	-	-	-	-	4.7%	-	-	-	-
8.9×10^4^		-	-	-	-	-	-	-	0.0%	-	-	-	-
1.2×10^5^		-	-	-	-	19%	15%	39%	-	-	-	-	-
1.8×10^5^		-	-	-	-	-	-	-	2.1%	-	-	-	-
3.6×10^5^		-	-	-	-	-	-	-	5.1%	-	-	-	-
3.8×10^5^		-	-	-	-	17%	5.4%	14%	-	-	-	-	-
7.2×10^5^		-	-	-	-	-	-	-	12%	-	-	-	-
1.4×10^6^		-	-	-	-	-	-	-	12%	-	-	-	-
2.9×10^6^		-	-	-	-	-	-	-	2.6%	-	-	-	-
PANSORBIN cells (cells/mL)	PBMC Lot	#D2.1	#D2.2	#D2.3	#D2.4	#D2.5	#D2.6	#D2.7	#D2.8	#D5. 01–04	#D5. 05–08	#D5. 09–12	#D5. 13–16
1.0×10^4^		-	-	-	-	6.5%	-	-	-	-	-	-	-
3.2×10^4^		-	-	-	-	12%	19%	6.2%	-	-	-	-	-
1.0×10^45^		-	-	-	-	18%	6.3%	13%	-	-	-	-	-
3.2×10^5^		-	-	-	-	-	14%	-	-	-	-	-	-

LPS, lipopolysaccharide; HKSA, heat-killed *Staphylococcus aureus*; PBMC, peripheral blood mononuclear cells.

- not determined.

**Fig 5:**
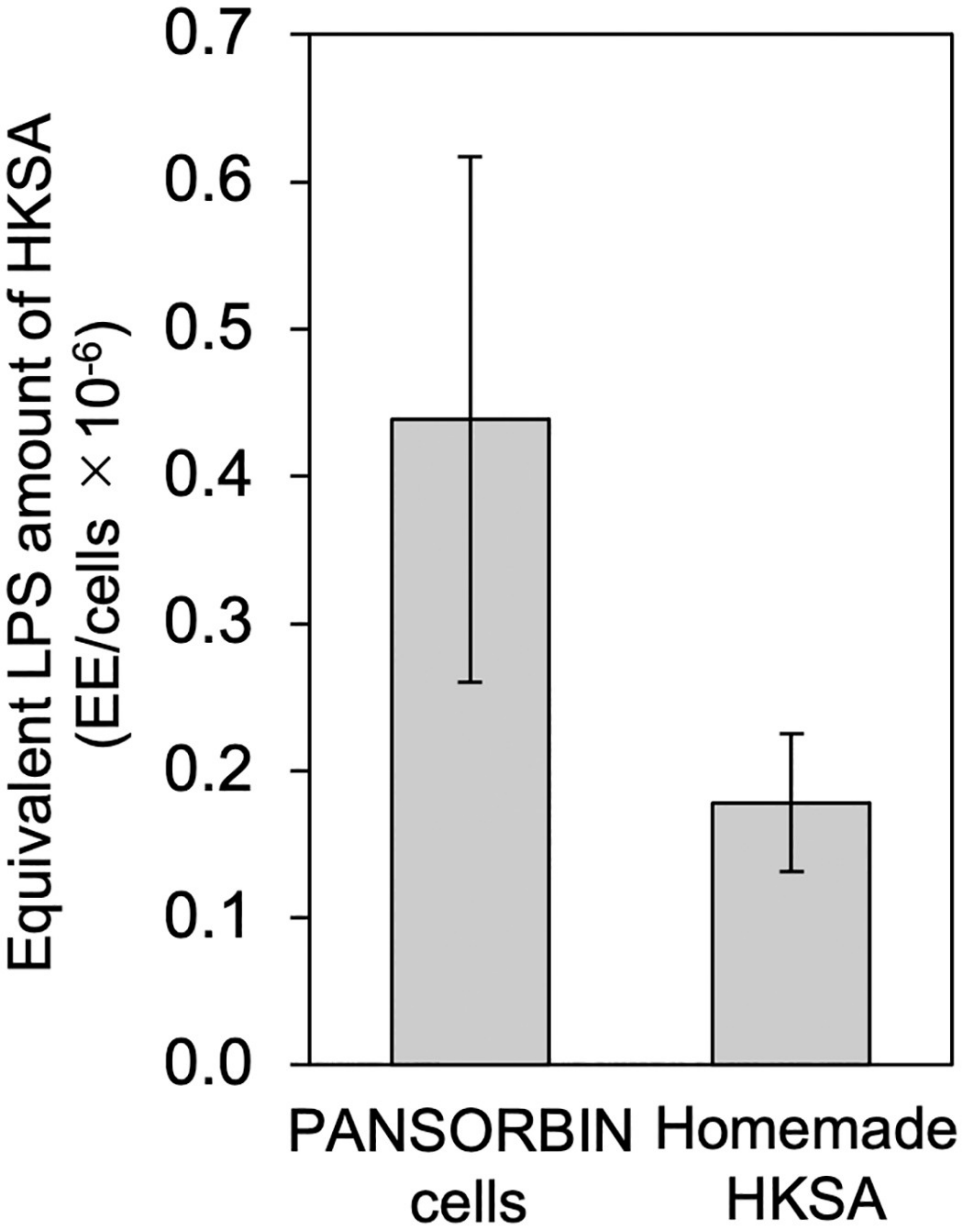
Average equivalent LPS amount of heat-killed *Staphylococcus aureus* cells. The IL-6 concentration from four lots of 2-d-old PBMCs (#D2.5–#D2.8) stimulated with heat-killed *Staphylococcus aureus* (HKSA) was converted using the 4-parameter logistic regression curve relating lipopolysaccharide (LPS) and IL-6 concentrations. Bars represent standard deviations. EE: equivalent endotoxin unit.

To evaluate reproducibility, the equivalent LPS amount of HKSA was calculated using the relationship between IL-6 and HKSA and LPS concentrations in PBMC lots #D2.5, #D2.6, #D2.7, and #D2.8 (Fig 5 and S1 File). The averaged equivalent LPS amounts of PANSORBIN cells and homemade HKSA were (0.44 ± 0.178) × 10^−6^ and (0.18 ± 0.047) × 10^−6^ EE/cells, respectively, which CV values were calculated to be 40.66% and 26.47%. As the HKSA concentration used in the MAT assays increased, the equivalent LPS amount of HKSA tended to decrease (S8 Fig and S1 File).

## Discussion

In this study, to elucidate the effects of cell freshness on pyrogen detection, the serum-free MAT with LBP was evaluated to ensure the reliable detection of pyrogens. The serum-free MAT exhibited high sensitivity to LPS in the presence of LBP (Fig 1, S1 and S6 Figs). The LODs of the serum-free MAT with LBP showed slight differences ranging from 0.001 to 0.008 EU/mL, except for PBMC lot #D2.4, which showed a higher LOD of 0.032 EU/mL. Jaekal *et al*. [26] reported that PBMCs from three out of 27 individuals (11.1%) showed low sensitivity to LPS. In the case of this study, only lot #D2.4 exhibited low sensitivity among eight PBMC lots (12.5%), a ratio comparable to the reported frequency of low-sensitivity individuals. In the absence of LBP, the reactivity to LPS decreased by a factor of 38.6 (Fig 2 and S2 Fig), indicating that LBP functions as an enhancer for LPS detection.

In the presence of LBP, serum-free MAT showed sufficient LODs (0.001–0.008 EU/mL; Fig 1, S1 and S6 Figs) because the serum-based MAT reported by Solati *et al*. [22] had an LOD of 0.002–0.004 EU/mL for LPS. Moreover, the present serum-free MAT showed a sufficient LOD for HKSA (S4 Fig), as the serum-based MAT by Solati *et al*. [22] showed that the LOD of HKSA was approximately 0.000014% of PANSORBIN cells, which corresponds to 3.4×10^4^ cells/mL. Therefore, the serum-free MAT successfully detected both LPS and HKSA in the presence of LBP.

Serum-free MAT used LBP, which did not enhance reactivity to HKSA (S5 Fig) since LBP is an LPS-reactive molecule in the blood [14]. Certain serum components may improve the sensitivity and reactivity of serum-free MAT because the serum affects the sensitivity and reactivity of HKSA [27]. Therefore, further research on using additives in serum-free MAT is required.

To examine the effect of cell freshness on MAT, the LOD of LPS was determined using 5-d-old PBMCs cultured in serum-free MAT in the presence of LBP (Fig 3). The LODs of LPS in 5-d-old PBMCs were slightly higher than those in 2-d-old PBMCs (S1 and S7 Figs), and the 3.1-fold difference in reactivity did not affect the 2- and 5-d-old PBMCs (Fig 4). These insights suggest that the sensitivity of PBMCs decreases gradually rather than sharply.

When MAT is performed for pharmaceutical injections, dilution is crucial to prevent interference with the response to pyrogens [12]. A low LOD is desirable for MAT because a high dilution factor is necessary, especially for injectables that can cause considerable interference. An LOD of 0.03 EU/mL LPS was reported, demonstrating feasibility for a blood-derived product [27]. This LOD was marginal for 5-d-old PBMCs (S7 Fig), although it was a sufficiently low LOD when using 2-d-old PBMCs (S1 Fig). Therefore, we concluded that the 2-d-old PBMCs were sufficiently fresh for use in MAT.

PBMCs are recommended for use in the MAT procedure described in Chapter 2.6.30 of the Ph. Eur. [12], to be used within 4 h of collection. This study demonstrates that PBMCs isolated from 2-d-old, out-of-specification donated whole blood supplied by the JRCS, can be used for MAT. PBMCs from donated whole blood offer the advantage of a low risk of infection [28]. However, there is a disadvantage of lot-to-lot variability in the sensitivity, such as in PBMC lot #D2.4 (S1 Fig). Further research on the acceptance criteria for blood samples is required to eliminate low-sensitivity PBMCs.

In addition to PBMC sensitivity, it is important to check the accuracy and reproducibility of MAT assays. Regarding the accuracy, Chapter 2.6.30 of Ph. Eur. [12] recommends a CV value of < 20%. The serum-free MAT generally showed CV values that were less than 20% (Table 1). This suggests that the serum-free MAT yielded accurate results. Because the CV values of 5-d-old PBMC were larger than those of 2-d-old PBMC, using old blood can affect the accuracy of the assay. For reproducibility, the equivalent LPS amounts of PANSORBIN cells and homemade HKSA were estimated at 0.44 × 10^−6^ and 0.18 × 10^−6^ EE/cells with CVs of 40.66% and 26.47%, based on four PBMC lots #D2.5, #D2.6, #D2.7, and #D2.8 (Fig 5). Because the dose-response relationships of HKSA and LPS are different (S8 Fig), it is challenging to directly compare the equivalent LPS amount for HKSA, which may increase the CV of the results. Considering that Chapter 2.6.30 of the Ph. Eur. requires endotoxin spike recoveries within the range of 50–200% [12], the observed CVs of 40.66% and 26.47% are fundamentally acceptable. Nevertheless, additional studies are required to enhance the reproducibility of the serum-free MAT.

In conclusion, serum-free MAT showed sufficient sensitivity to LPS, with an LOD of 0.001–0.008 EU/mL in the presence of LBP. Using this serum-free MAT, comparing the reactivity between 2- and 5-d-old PBMCs suggests that 2-d-old PBMCs are sufficiently fresh to serve as a cell source for MAT to detect LPS. These findings provide valuable insights that may contribute to the development of MAT approaches.

## Supporting information

S1 File Statistical details. Data were analyzed using Microsoft Excel for Mac version 16.78.3 and R version 4.3.0.
(XLSX)

S1 Fig Detection of lipopolysaccharide (LPS) by serum-free monocyte-activation test using 2-d-old peripheral blood mononuclear cells (PBMCs) in the presence of LPS-binding protein. The interleukin-6 (IL-6) production in the culture supernatant of four PBMC lots (#D2.1–D2.4) was plotted. Dotted lines and numbers represent threshold IL-6 levels at the limit of detection (LOD). The asterisks represent the LODs of the assay. EU, endotoxin unit.
(TIF)

S2 Fig Detection of lipopolysaccharide (LPS) by serum-free monocyte-activation tests using 2-d-old peripheral blood mononuclear cells (PBMCs) in the absence of LPS-binding protein. The interleukin-6 (IL-6) production in the culture supernatant of four PBMC lots (#D2.1–D2.4) was plotted. Dotted lines and numbers represent threshold IL-6 levels at the limit of detection (LOD). The asterisks represent the LODs of the assay. LPS, lipopolysaccharides; EU, endotoxin unit.
(TIF)

S3 Fig Comparison of the reactivities to lipopolysaccharide (LPS) on a serum-free monocyte-activation test in the presence and absence of LPS-binding protein (LBP). Using the data from three PBMC lots (#D2.1–D2.4), the interleukin-6 (IL-6) productions in culture supernatant above the limit of detection were plotted. Dashed dotted and short dashed lines represent linear regressions in the presence and absence of LBP, respectively. Regression equations are provided next to the regression lines. LBP (+), in the presence of 100 ng/mL LBP; LBP (−), in the absence of LBP; EU, endotoxin unit.
(TIF)

S4 Fig Detection of heat-killed *Staphylococcus aureus* (HKSA) by serum-free monocyte-activation test. The interleukin-6 (IL-6) productions in the culture supernatant of three PBMC lots (#D2.5–D2.7) were plotted. Dotted lines and numbers represent threshold IL-6 levels at the limit of detection (LOD). The asterisks represent the LODs of the assay.
(TIF)

S5 Fig Comparison of the reactivities to heat-killed *Staphylococcus aureus* (HKSA) on serum-free monocyte-activation tests in the presence and absence of LPS-binding protein (LBP). Using data from PBMC lot #D2.8, interleukin-6 (IL-6) production in the culture supernatant above the limit of detection was plotted. Dashed dotted and short dashed lines represent linear regression in the presence and absence of LBP, respectively. The regression equations are provided next to the regression lines. LBP (+), in the presence of 100 ng/mL LBP; LBP (−), in the absence of LBP.
(TIF)

S6 Fig Detection of lipopolysaccharide (LPS) by serum-free monocyte-activation test in the presence of LPS-binding protein test using 2-d-old peripheral blood mononuclear cells (PBMCs) used for detection of heat-killed *Staphylococcus aureus* (HKSA). The interleukin-6 (IL-6) production in the culture supernatant of four PBMC lots (#D2.5–D2.8) was plotted. Dotted lines and numbers represent threshold IL-6 levels at the limit of detection (LOD). The asterisks represent the LODs of the assays. EU, endotoxin unit.
(TIF)

S7 Fig Detection of lipopolysaccharide (LPS) by serum-free monocyte-activation test using 5-d-old peripheral blood mononuclear cells (PBMCs) in the presence of LPS-binding protein. The interleukin-6 (IL-6) production in the culture supernatant of four experiments of 5-d-old using 16 lots of PBMCs (#D5.01–04, #D5.05–08, #D5.09–12, and #D5.13–16) is plotted. Dotted lines and numbers represent threshold IL-6 levels at the limit of detection (LOD). The asterisks represent the LODs of the assays. EU, endotoxin unit.
(TIF)

S8 Fig Relationships between the concentration of heat-killed Staphylococcus aureus (HKSA) used in the MAT assay and the equivalent lipopolysaccharide amount of HKSA. Bars represent standard deviations. EE, equivalent endotoxin unit; LPS, lipopolysaccharides; MAT, monocyte-activation test; PBMC, peripheral blood mononuclear cells.
(TIF)
