# Supplementary figures and images for: Evaluation of the effect of cell freshness on pyrogen detection using a serum-free monocyte-activation test
Source: PLoS One. 2024 Dec 30;19(12):e0316203. doi: 10.1371/journal.pone.0316203 (PMC11684592; doi:10.1371/journal.pone.0316203)

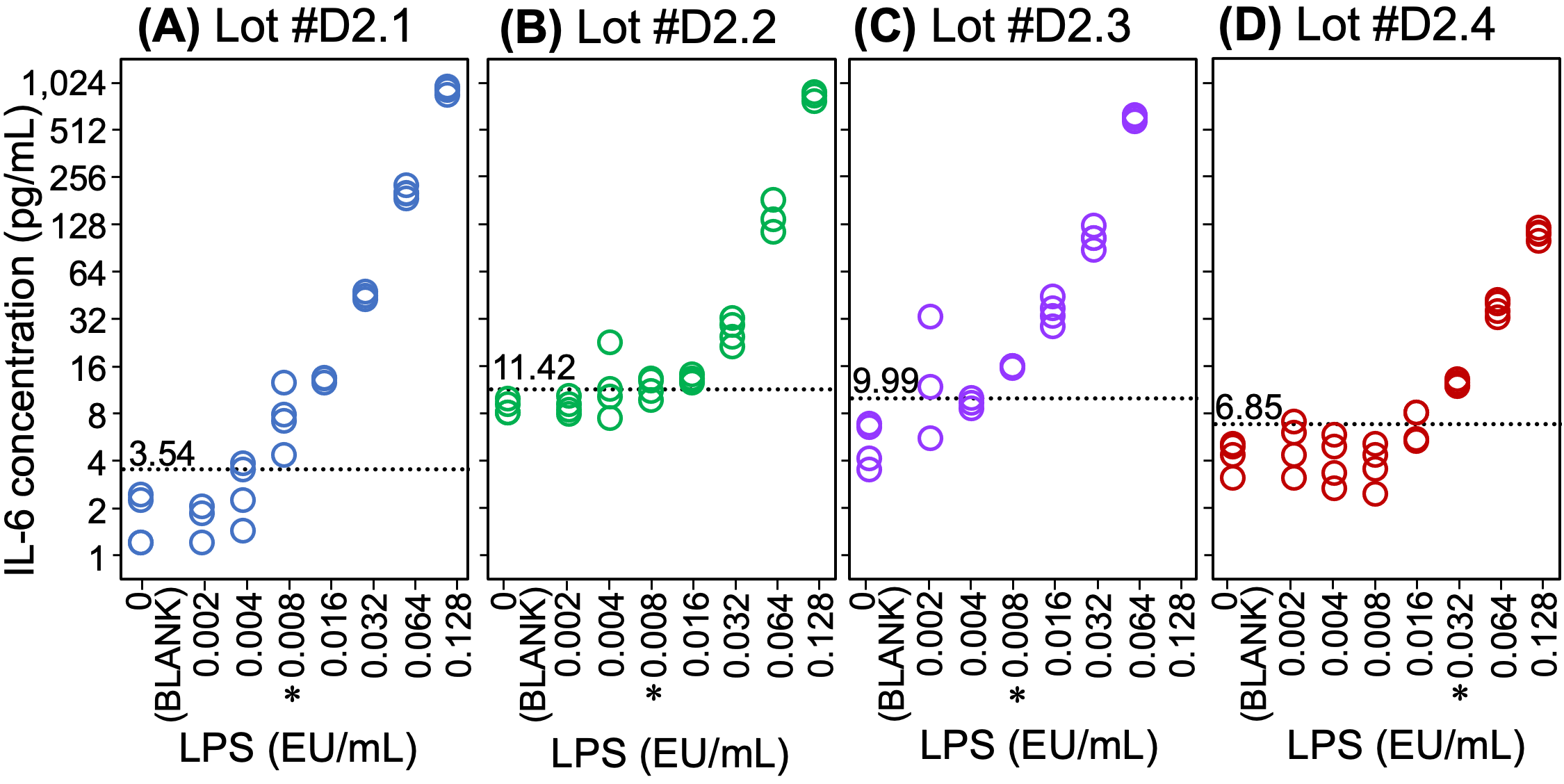

Supplement: S1 Fig — The interleukin-6 (IL-6) production in the culture supernatant of four PBMC lots (#D2.1–D2.4) was plotted. Dotted lines and numbers represent threshold IL-6 levels at the limit of detection (LOD). The asterisks represent the LODs of the assay. EU, endotoxin unit. (TIF) [file pone.0316203.s002.tif]

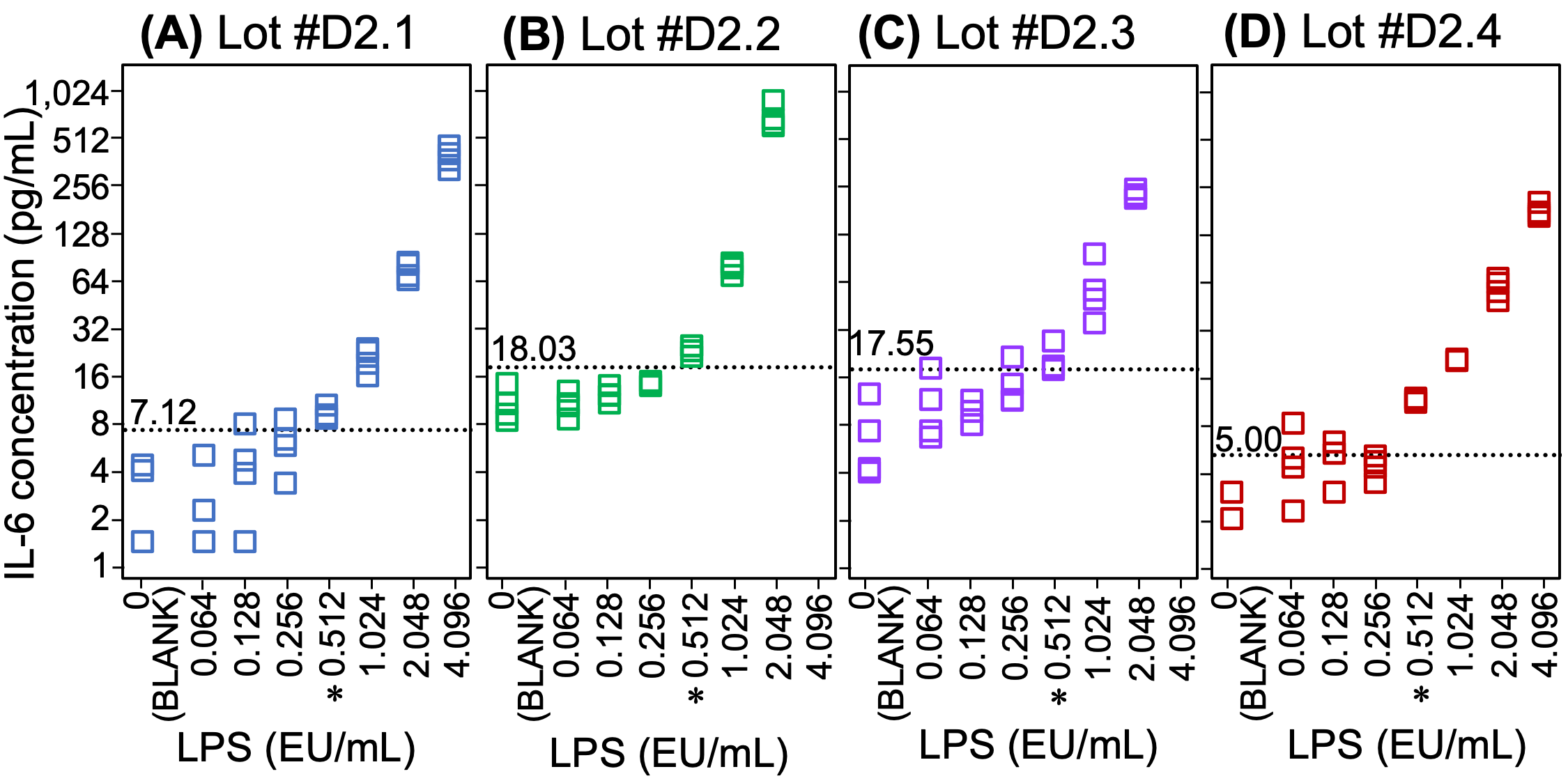

Supplement: S2 Fig — The interleukin-6 (IL-6) production in the culture supernatant of four PBMC lots (#D2.1–D2.4) was plotted. Dotted lines and numbers represent threshold IL-6 levels at the limit of detection (LOD). The asterisks represent the LODs of the assay. LPS, lipopolysaccharides; EU, endotoxin unit. (TIF) [file pone.0316203.s003.tif]

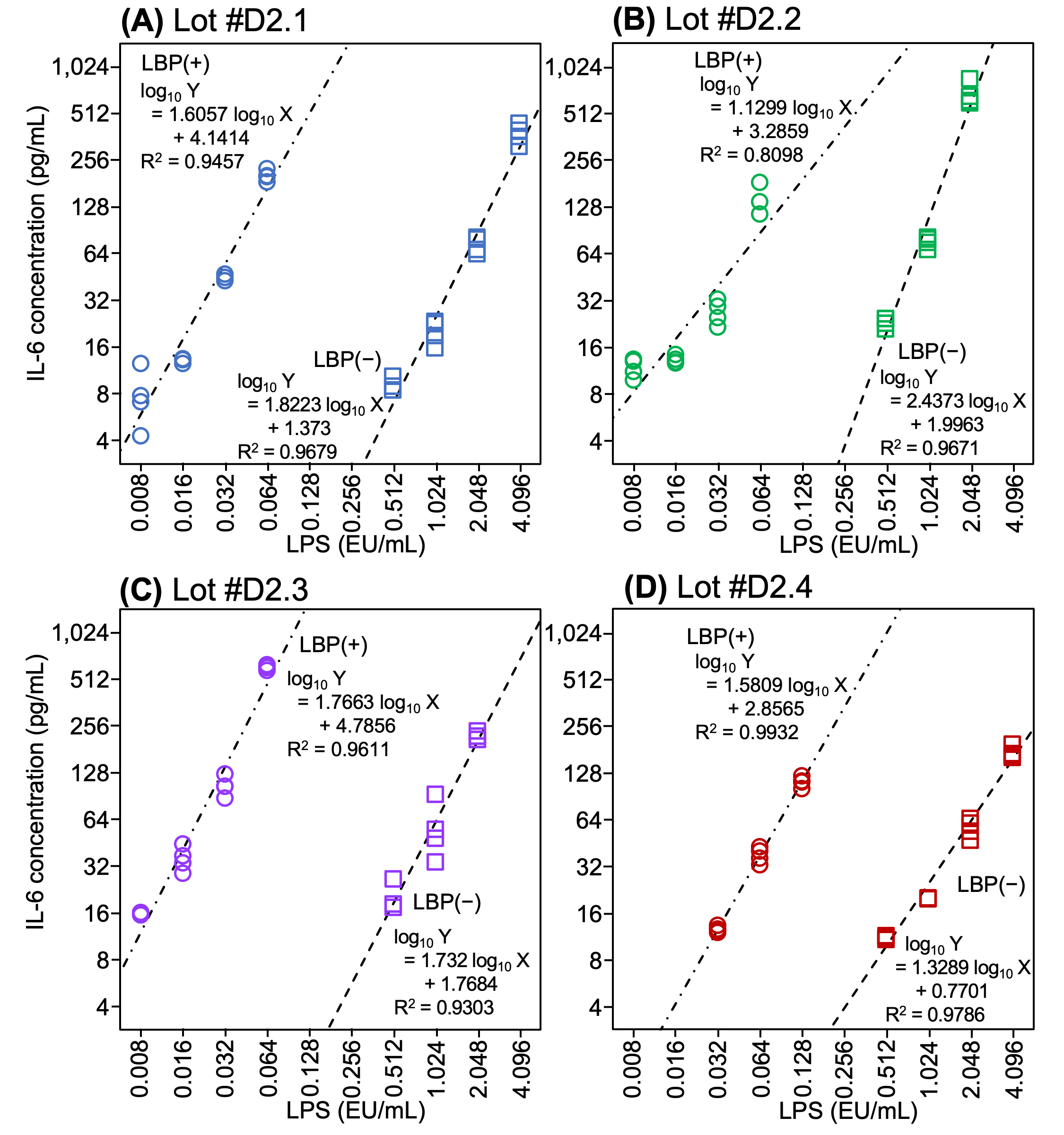

Supplement: S3 Fig — Using the data from three PBMC lots (#D2.1–D2.4), the interleukin-6 (IL-6) productions in culture supernatant above the limit of detection were plotted. Dashed dotted and short dashed lines represent linear regressions in the presence and absence of LBP, respectively. Regression equations are provided next to the regression lines. LBP (+), in the presence of 100 ng/mL LBP; LBP (−), in the absence of LBP; EU, endotoxin unit. (TIF) [file pone.0316203.s004.tif]

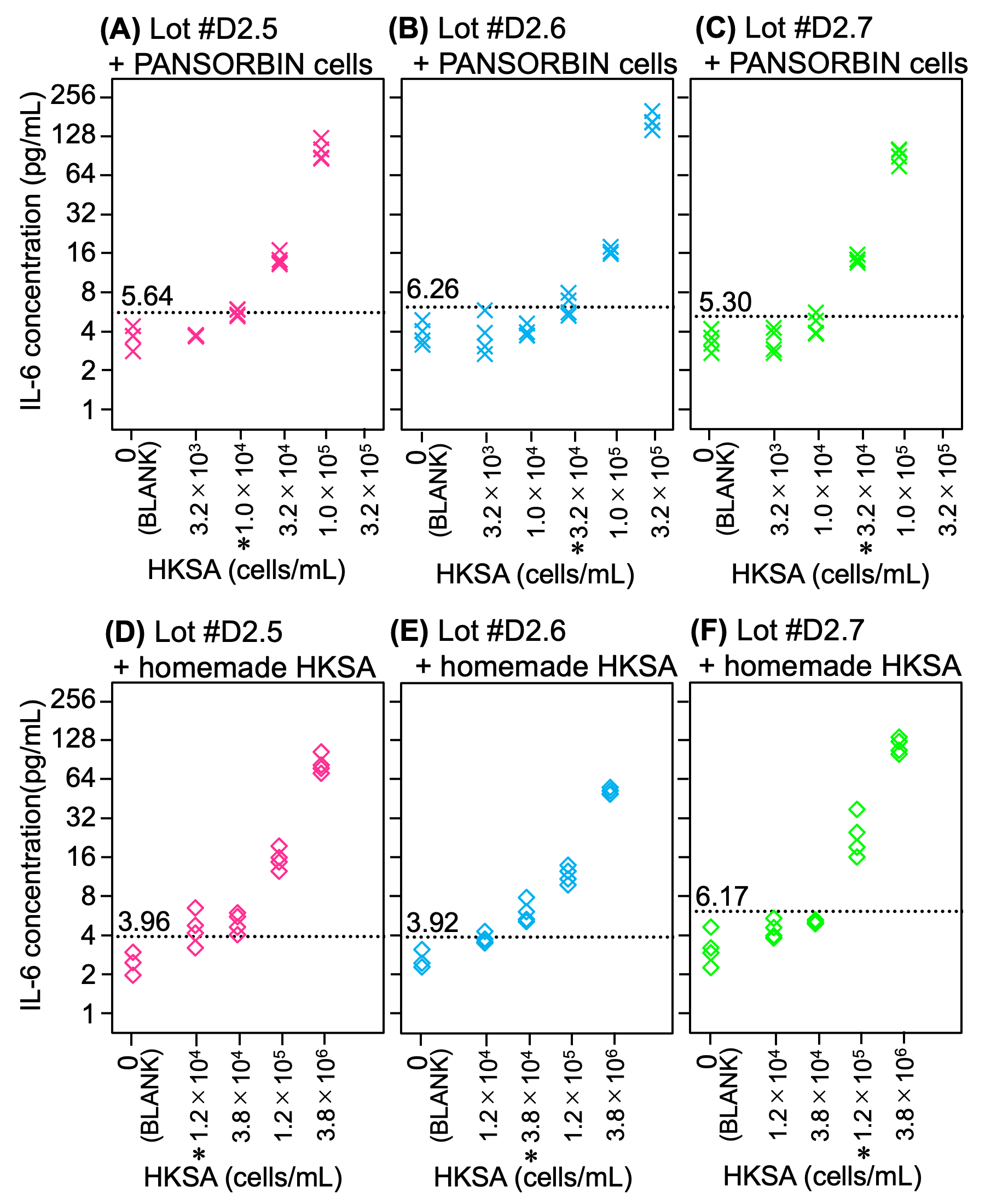

Supplement: S4 Fig — The interleukin-6 (IL-6) productions in the culture supernatant of three PBMC lots (#D2.5–D2.7) were plotted. Dotted lines and numbers represent threshold IL-6 levels at the limit of detection (LOD). The asterisks represent the LODs of the assay. (TIF) [file pone.0316203.s005.tif]

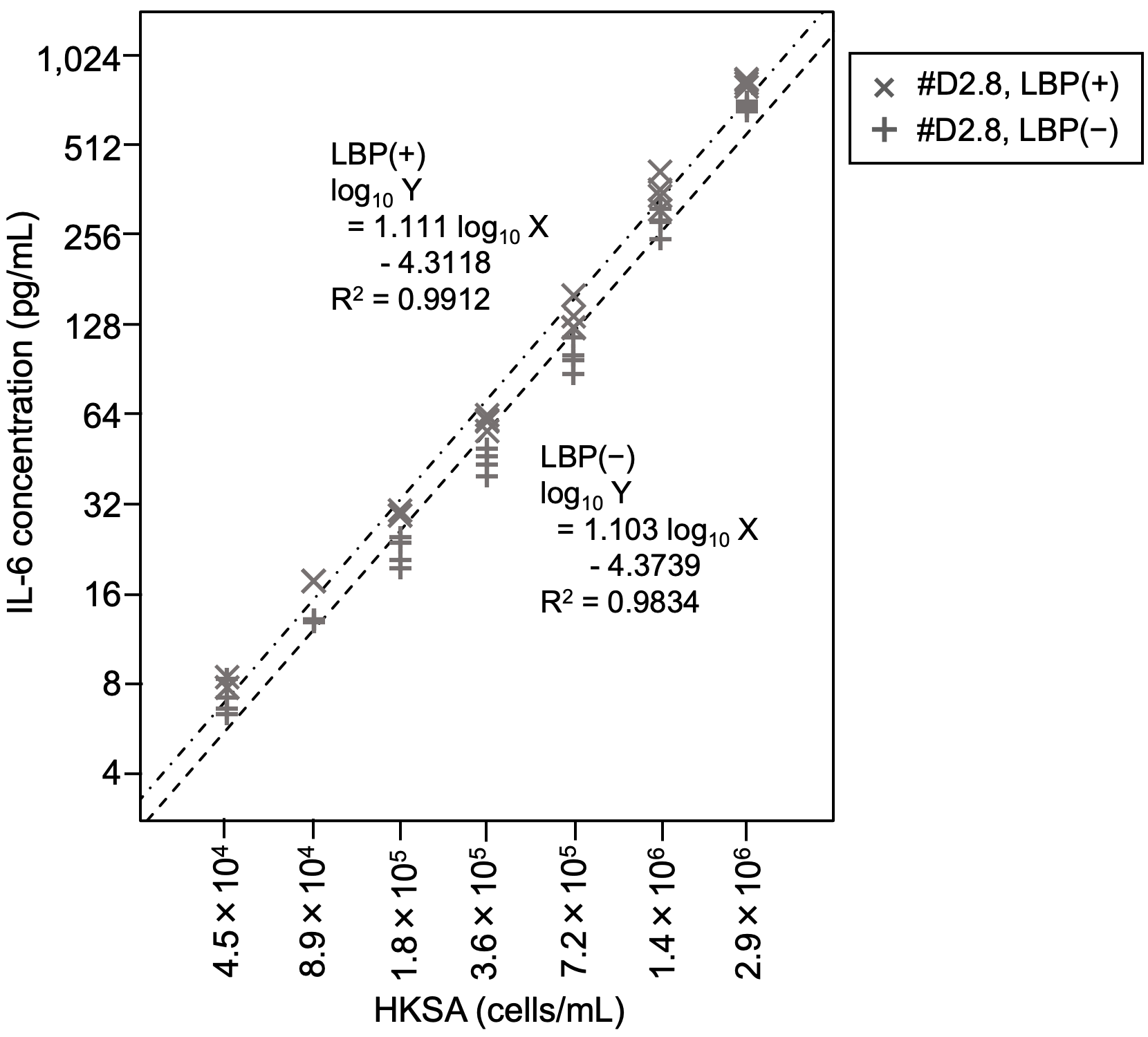

Supplement: S5 Fig — Using data from PBMC lot #D2.8, interleukin-6 (IL-6) production in the culture supernatant above the limit of detection was plotted. Dashed dotted and short dashed lines represent linear regression in the presence and absence of LBP, respectively. The regression equations are provided next to the regression lines. LBP (+), in the presence of 100 ng/mL LBP; LBP (−), in the absence of LBP. (TIF) [file pone.0316203.s006.tif]

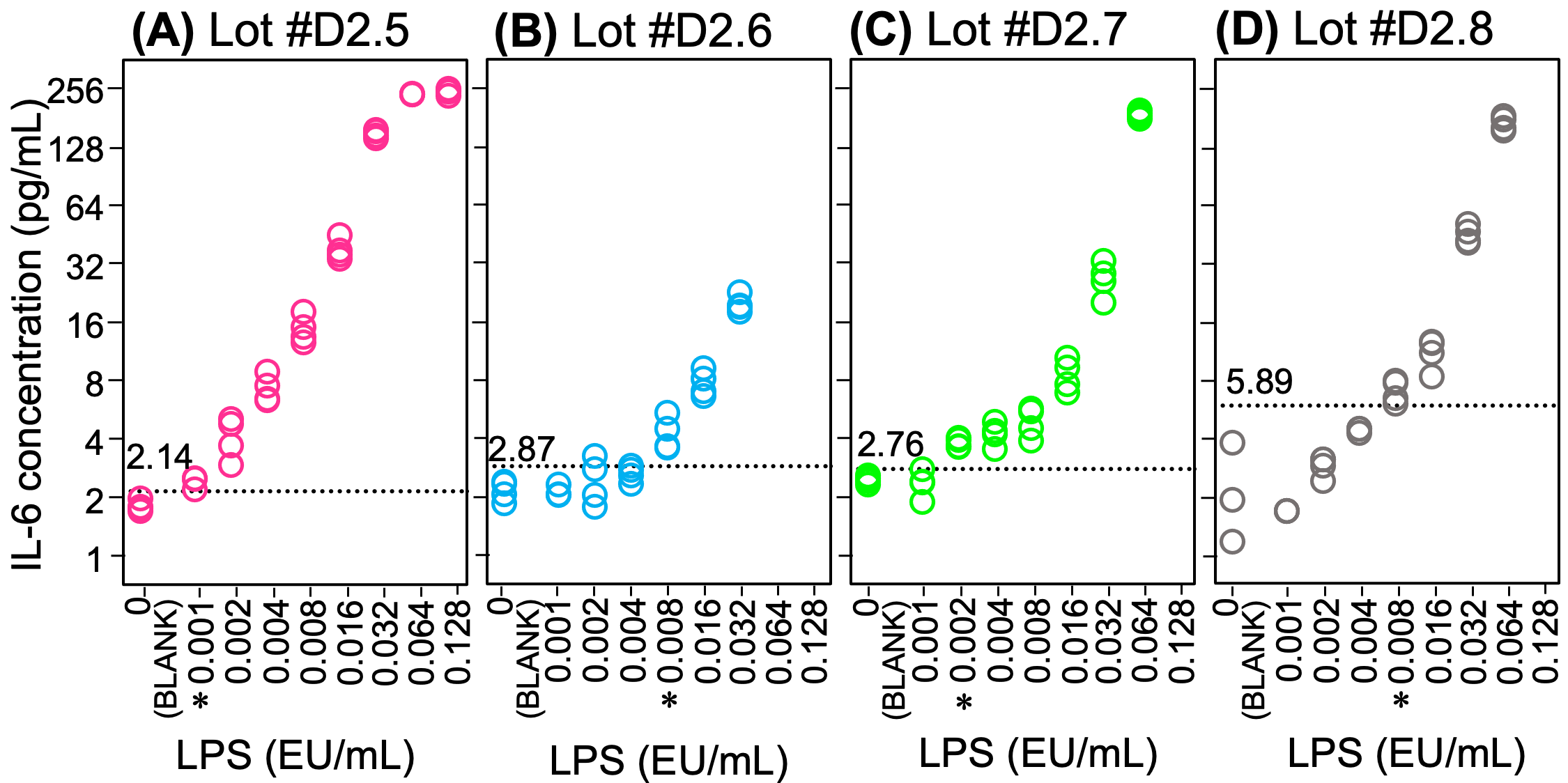

Supplement: S6 Fig — The interleukin-6 (IL-6) production in the culture supernatant of four PBMC lots (#D2.5–D2.8) was plotted. Dotted lines and numbers represent threshold IL-6 levels at the limit of detection (LOD). The asterisks represent the LODs of the assays. EU, endotoxin unit. (TIF) [file pone.0316203.s007.tif]

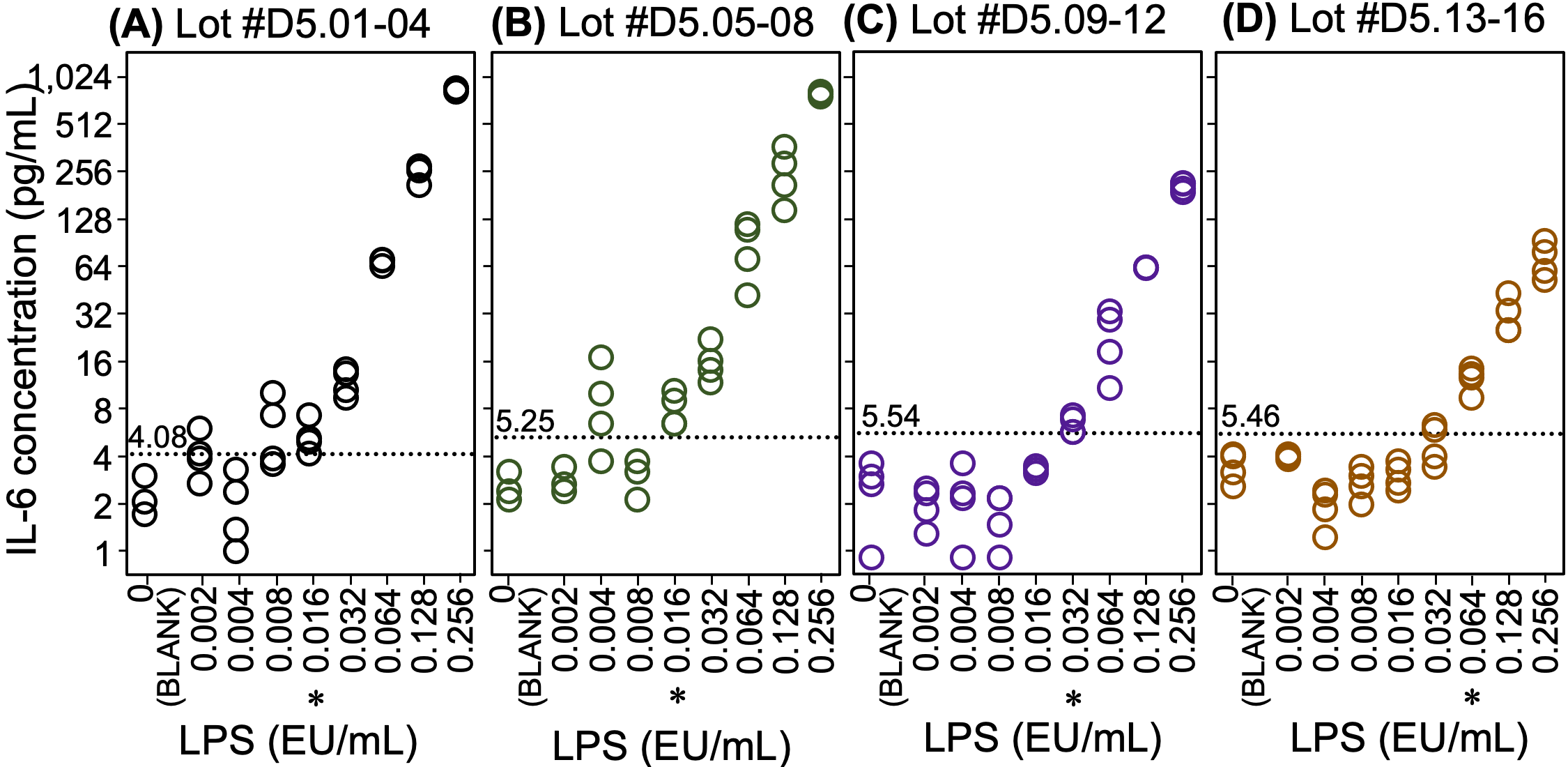

Supplement: S7 Fig — The interleukin-6 (IL-6) production in the culture supernatant of four experiments of 5-d-old using 16 lots of PBMCs (#D5.01–04, #D5.05–08, #D5.09–12, and #D5.13–16) is plotted. Dotted lines and numbers represent threshold IL-6 levels at the limit of detection (LOD). The asterisks represent the LODs of the assays. EU, endotoxin unit. (TIF) [file pone.0316203.s008.tif]

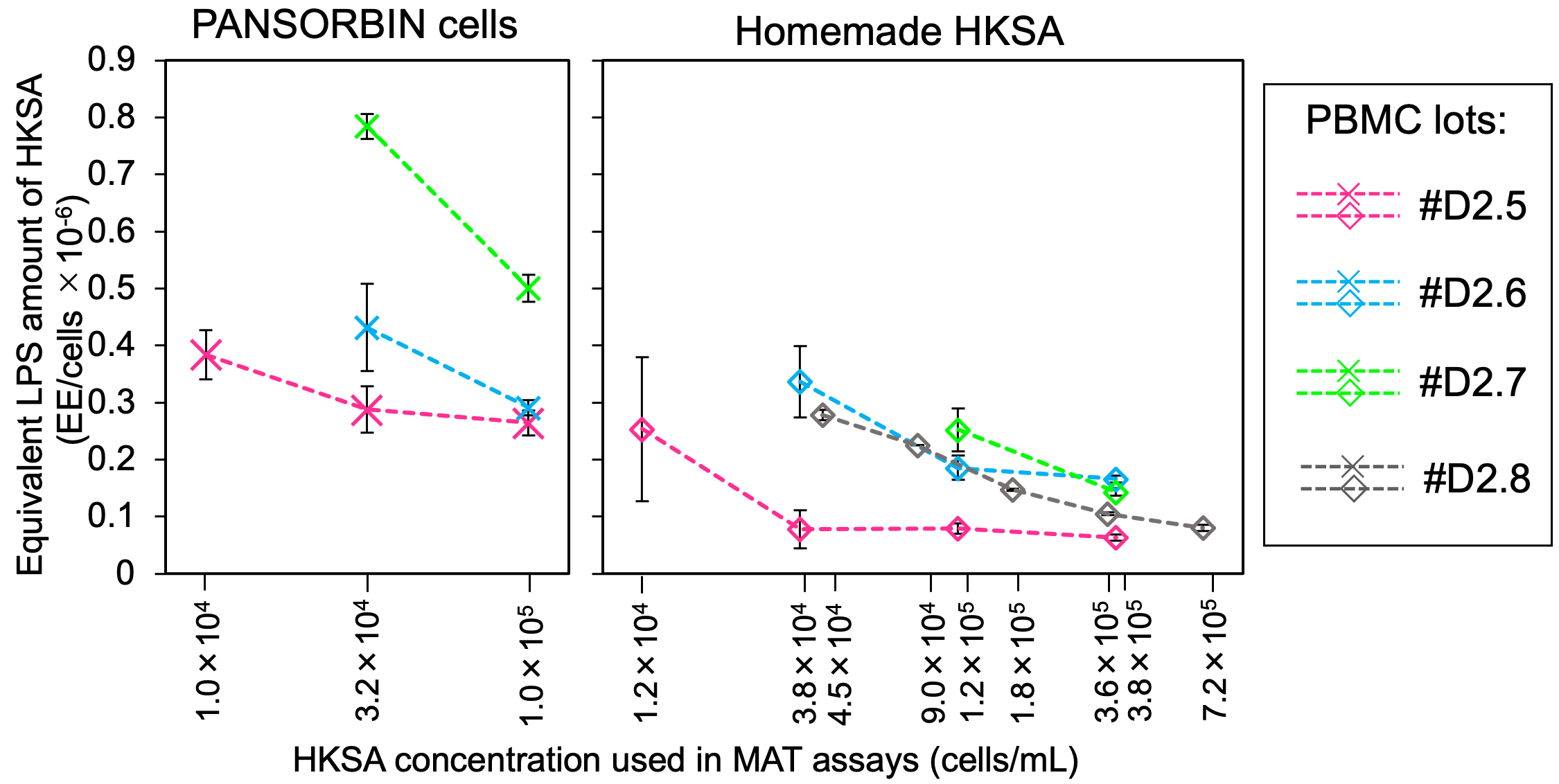

Supplement: S8 Fig — Bars represent standard deviations. EE, equivalent endotoxin unit; LPS, lipopolysaccharides; MAT, monocyte-activation test; PBMC, peripheral blood mononuclear cells. (TIF) [file pone.0316203.s009.tif]
